# Finite Element Simulation and Microstructural Evolution Investigation in Hot Stamping Process of Ti6Al4V Alloy Sheets

**DOI:** 10.3390/ma17061388

**Published:** 2024-03-18

**Authors:** Mingjia Qu, Zhengwei Gu, Xin Li, Jianbo Wang, Ge Yu, Lingling Yi

**Affiliations:** 1 State Key Laboratory of Automobile Materials, Jilin University, Changchun 130025, China; qumj21@mails.jlu.edu.cn (M.Q.); li_xin@jlu.edu.cn (X.L.); yuge@jlu.edu.cn (G.Y.); yill20@mails.jlu.edu.cn (L.Y.); 2 Department of Materials Science and Engineering, Jilin University, Changchun 130025, China; 3 CRRC Changchun Railway Vehicles Co., Ltd., Maglev Technology Institude, Changchun 130021, China

**Keywords:** titanium alloy, hot stamping, response surface methodology, finite element simulation

## Abstract

Titanium alloy hot stamping technology has a wide range of application prospects in the field of titanium alloy part processing due to its high production efficiency and low manufacturing cost. However, the challenges of forming titanium alloy parts with large depths and deformations have restricted its development. In this study, the hot stamping process of a Ti6Al4V alloy box-shaped part was investigated using ABAQUS 2020 software. The thermodynamic properties of a Ti6Al4V alloy sheet were explored at different temperatures (400 °C, 500 °C, 600 °C, 700 °C, 800 °C) and different strain rates (0.1 s^−1^, 0.05 s^−1^, 0.01 s^−1^). In addition, the influence law of hot stamping process parameters on the minimum thickness of the formed part was revealed through the analysis of response surface methodology (RSM), ultimately obtaining the optimal combination of process parameters for Ti6Al4V alloy hot stamping. The experimental results of the hot stamping process exhibited a favorable correlation with the simulated outcomes, confirming the accuracy of the numerical simulation. The study on the microstructure evolution of the formed parts showed that grain refinement strengthening occurred in the part with large deformation, and the formed box-shaped parts exhibited a uniform and fine microstructure overall, demonstrating high forming quality. The achievements of the work provide important guidance for the fabrication of titanium alloy parts with large depths and deformations used in heavy industrial production.

## 1. Introduction

Titanium alloy has been widely used in aerospace field by virtue of its excellent mechanical properties. Due to its high specific strength and corrosion resistance [1,2], titanium alloy exhibits broad prospects in lightweight applications [3]. As a consequence, individuals are progressively extending their applications to the transportation sector [4]. Therefore, there is an urgent need for an efficient and cost-effective specialized forming method for titanium alloy. Owing to the poor plasticity and high springback of titanium alloy at room temperature [5], the forming of titanium alloy sheets is typically conducted at high temperatures. Common conventional hot forming processes for titanium alloy include isothermal hot pressing [6], superplastic forming [7], hot stretch forming [8], and so on. But these processes require the sheet to maintain a high and constant temperature during deformation, which means that the forming tool needs to be heated, which increases the manufacturing cost and reduces productivity. In recent years, a hot stamping technology for titanium alloy sheets has been proposed, which has the advantages of short production cycles, low manufacturing costs, and less equipment requirements. The in-depth exploration of hot stamping technology for titanium alloys holds significant implications for reducing manufacturing costs and enhancing production.

To elucidate the deformation behavior of titanium alloy during the hot stamping process, extensive research has been undertaken to investigate its high-temperature mechanical properties. Kopec et al. [9] analyzed the high-temperature mechanical properties of Ti6Al4V alloy and found significant differences in the mechanical behavior and deformation mechanisms of titanium alloy at different temperatures, with significant strain hardening occurring at temperatures lower than 600 °C. Liu et al. [10] characterized the interfacial heat transfer coefficient of titanium alloy during hot stamping and found that titanium alloy cools down significantly faster than steels and other materials during the hot stamping process. Gao et al. [11] investigated the high-temperature mechanical properties of Ti6Al4V alloy sheets by hot tensile experiments in the temperature range of 650–750 °C and compared the prediction accuracy of different constitutive models. Liu et al. [12] studied the deformation behavior of Ti6Al4V alloy in the temperature range of 800–890 °C. However, most of the current studies on titanium alloy hot deformation behavior focus on the field of isothermal hot forming, while the hot stamping of titanium alloys is accompanied by a relatively significant temperature change, so the study of the thermomechanical properties of titanium alloys containing a large temperature range is necessary.

Currently, there is a broader application of hot stamping technology for titanium alloy parts with smaller deformation, and there is a lack of research on the hot stamping of titanium alloy parts with large depths and deformations amounts [13,14]. Yang et al. [15] discovered that when performing conventional hot stamping with titanium alloy sheet material, the obtained forming depth was only 13 mm. However, in multi-layer sheet hot stamping, a hemispherical part with a forming depth of 33 mm could be obtained. But the increased complexity of the processing technology undoubtedly raises manufacturing costs. In contrast, in the field of hot stamping with materials such as aluminum and steel, finite element simulation methods have been widely applied to improve forming quality. Yi et al. [16] used PAM-STAMP 2019 software to simulate the formation of a 5083 aluminum alloy skin, analyzed the influence of forming process parameters on the quality of the parts based on response surface methodology (RSM), and successfully optimized the processing parameters. Tang et al. [17] combined numerical simulation and experiments to optimize the preforming amount of 22MnB5 steel, resulting in improved forming quality and more uniform thickness distribution in final formed part. Neumann et al. [18] used Abaqus 2020 software to simulate the hot stamping process of sheet metals into a W-shape profile and suggested that the thermo-micromechanical model is more consistent with the final shape and residual stress state of hot stamped parts. However, the optimization of process parameters for the hot stamping of titanium alloys via combined numerical simulation is not yet available.

In this paper, the mechanical properties of Ti6Al4V alloy under a temperature range of 400–800 °C and strain rate range of 0.1–0.01 s^−1^ were investigated through hot tensile tests. Simultaneously, the shape of the sheets was optimized by ABAQUS 2020 finite element software, and the effects of entrance fillet radius, blank-holder force, and friction coefficient on forming quality were investigated using RSM. By fitting regression equations, the optimal combination of forming process parameters was obtained, and parts meeting quality standards were successfully manufactured. Finally, the microstructural evolution in different regions of the formed parts was studied using scanning electron microscopy (SEM) and transmission electron microscopy (TEM), confirming the accuracy of the simulation and the feasibility of the process.

## 2. Materials and Experimental Details

### 2.1. Materials Model

**Figure 1:**
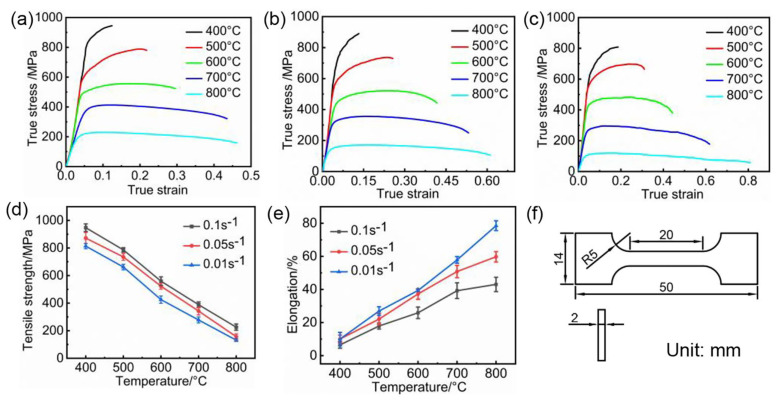
(**a**) Flow stress curves at strain rate of 0.1 s^−1^; (**b**) flow stress curves at strain rate of 0.05 s^−1^; (**c**) flow stress curves at strain rate of 0.01 s^−1^; (**d**) tensile strength at different temperatures and strain rates; (**e**) elongation at different temperatures and strain rates; (**f**) the size of tensile specimen.

The material used in this study is an industrial Ti6Al4V alloy (Baoji Titanium Industry Co., Ltd., Baoji, China) sheet with a thickness of 2 mm, and the chemical composition is shown in Table 1. Hot uniaxial tensile tests were performed in a WDW-100G high-temperature universal testing machine, manufactured by Jilin Guanteng Automation Technology Co., Ltd., Changchun, China. Forces and displacements were automatically collected during the tensioning process. The tensile specimens were cut into bone-shaped specimens of 50 mm length and 14 mm width as shown in Figure 1f. For better alignment with the mechanical properties of Ti6Al4V alloy during the hot stamping process, the specimens were preheated in a furnace at 800 °C for 2 min before the tensile experiment. Based on existing research, in the hot stamping forming process of titanium alloys, the deformation starting temperature is usually between 700 and 800 °C, and the temperature of the sheet material typically fluctuates around 400 °C at the end of formation [9,19], so the deformation temperatures were set to be 400 °C, 500 °C, 600 °C, 700 °C, and 800 °C, and according to the real hot stamping process, the strain rates were set to be 0.1 s^−1^, 0.05 s^−1^, and 0.01 s^−1^. Each specimen was heated to the set temperature, held for 3 min, and then subjected to the hot tensile test. The obtained hot tensile data are shown in Figure 1.

By comparing Figure 1a–c, it can be observed that with the rise in deformation temperature and the decrease in strain rate, the tensile strength of the material decreases, while the elongation increases, indicating the plasticity of material is enhanced. Figure 1d indicates that the influence of strain rate on elongation gradually diminishes as the deformation temperature decreases. Furthermore, as illustrated in Figure 1e, the tensile strength of the Ti6Al4V alloy varies significantly in the range of 500–600 °C, and the increase in tensile strength slows down when the temperature decreases to 400–500 °C. Such a phenomenon is attributed to the tensile strength of Ti6Al4V alloy at room temperature, which is typically in the range of 950–1200 MPa [20,21], and the tensile strength of the material under different strain rates at 400 °C is higher than 808 MPa. Consequently, as the deformation temperature continues to decrease, the plasticity of the titanium alloy becomes constrained. Therefore, the primary deformation of titanium alloy parts in the process of hot stamping should be conducted at temperatures above 400 °C.

### 2.2. Forming Processes and Parts

**Figure 2:**
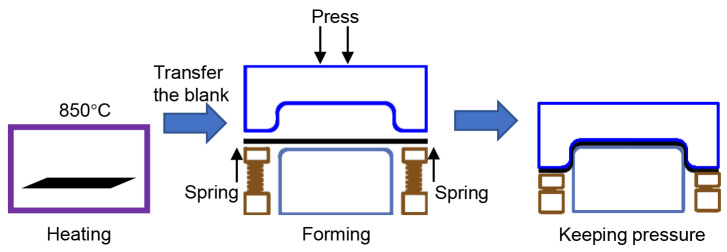
Schematic of the hot stamping process.

The processing steps for the hot stamping forming process of titanium alloy are illustrated in Figure 2. During the above process, the sheet material is firstly placed in a heating furnace (SX-G03173, Tianjin Zhonghuan Electric Furnace Co., Ltd., Tianjin, China) and kept for 2 min after the temperature has stabilized. Then, it is quickly removed from the furnace and transferred to room temperature dies for stamping. Then, using a four-column hydraulic press (YA32-5008, Xuzhou Dayi Forging & Press Equipment Co., Ltd., Xuzhou, China), Before the forming operation, the surface of the sheet is polished smooth and coated evenly with nano-scale boron nitride powder to prevent oxidation and provide lubrication. High-temperature grease is applied to surface of dies to reduce the friction coefficient. Due to the interfacial heat transfer coefficient of titanium alloy being much higher than 22MnB5 steel and other common hot stamping steel, as a result, the cooling rate of titanium alloy is typically excessively rapid during the hot stamping process. Therefore, conventional cooling water channels become irrelevant, so dies are not designed for cooling water channels. The material of the dies is H13 hot work tool steel; it possesses high strength, toughness, and good thermal fatigue stability, allowing it to withstand the cyclic thermal stresses caused by high and low temperature alternations [22].

**Figure 3:**
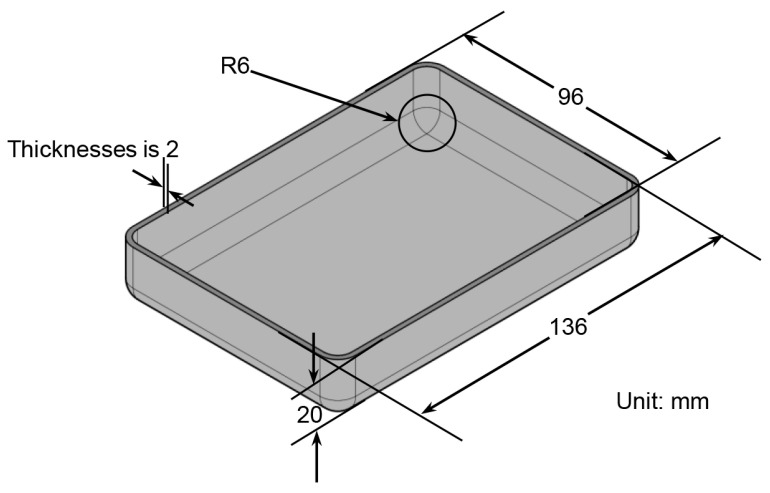
The geometric model of a titanium box-shaped part.

The dimensions of the titanium box-shaped parts are shown in Figure 3, and the size of the part is 136 mm × 96 mm × 20 mm, and the size of the internal fillet is 6 mm. Typically, the forming method of titanium alloy box-shaped parts with a certain depth consists of forming four sidewalls by hot bending and then connecting the sidewalls by welding at the corners of the box. However, this forming method is not only cumbersome but also results in poorer service performance in weakly formed areas such as the corners of the box.

In order to improve this drawback, this study adopts an integrated hot stamping method through the reasonable setting of die parameters and processing parameters: the stamping of box-shaped parts as a whole. In this process, the corners of the box-shaped parts are the location of the largest thinning amount. To ensure the mechanical performance of the formed component, the thickness in this area should be within a reasonable range.

## 3. Research Results for Hot Stamping and Forming of Case Parts

### 3.1. Numerical Simulation of Hot Stamping of Titanium Alloys

In the hot stamping process of titanium alloys, the plasticity of the Ti6Al4V alloy significantly diminishes with decreasing temperature, leading to reduced workability. The thinning ratio is an important parameter for evaluating the quality of hot forming. It is defined as the ratio of the difference in thickness before and after stamping to the original thickness of the sheet, which is an important indicator of the quality of sheet forming. It is generally recommended to control the thinning ratio of titanium alloy hot forming within 20%. Therefore, in the simulation of the hot stamping process described in this paper, the minimum thickness of the sheet should be higher than 1.6 mm. Consequently, it is necessary to plan the hot stamping forming process parameters properly to ensure the quality of the formed components meets the standards.

#### 3.1.1. Construction of the Finite Element Model

**Figure 4:**
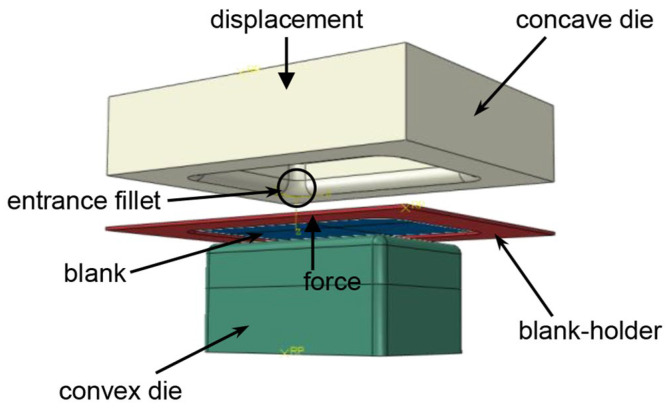
Finite element model of hot stamping process.

The finite element model of a blank and dies is shown in Figure 4. The dies comprise a convex die, a concave die, and a blank-holder; the blank is placed on the blank-holder, and as the concave die descends, the blank-holder is subjected to an elastic force to secure the blank between the blank-holder and concave die. The deformation process of hot stamping was simulated using ABAQUS 2020 software. In this work, hot tensile experimental data were input into Abaqus 2020 software as discrete data points. The initial temperature of the blank was set to 750 °C, while the temperature of the dies was set at 20 °C. The analysis step was set to dynamic, temperature displacement, and explicit. In addition, the blank was set as a shell, and the convex die, concave die, and blank-holder were set as rigid bodies. The blank was meshed by using a four-node thermally coupled doubly curved shell element (S4RT) with a mesh size of 2, and five integration points were set in the thickness direction [23]. The heat transfer coefficient between the blank and dies was 1700 W/(m^2^ °C) from Liu et al. [10]. According to the actual experimental setup, the blank-holder was supported by four industrial nitrogen gas springs, which can provide a fixed magnitude of elastic force when the blank-holder moves downwards. Therefore, in the simulation, the blank-holder was set to experience a constant upward force during the stamping process. The simulation of hot stamping was divided into two stages: the clamping stage and the forming stage. In clamping stage, the blank-holder applied the load, and blank was clamped between the concave die and blank-holder; in the forming stage, the concave die moved uniformly downward, driving the blank and blank-holder downward to complete the forming process.

#### 3.1.2. Optimization of Blank Shape

**Figure 5:**
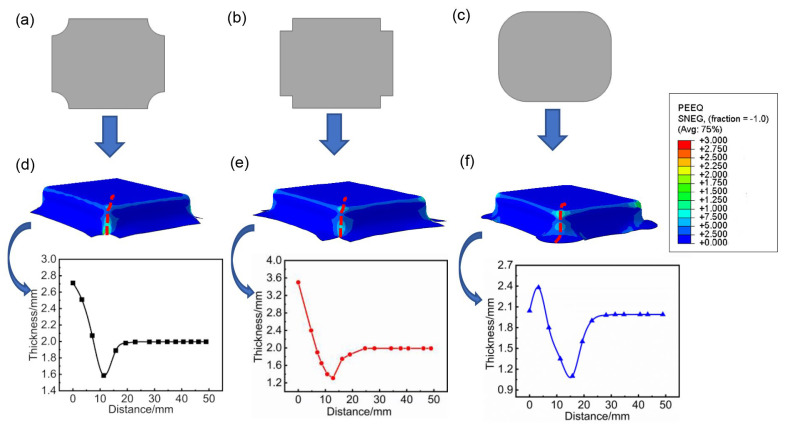
Shape and simulation results of different shape of crack stop incision: (**a**,**d**) inside concave curved; (**b**,**e**) right angled; (**c**,**f**) outward convex curved.

In the hot stamping process of titanium alloy, the rapid temperature fluctuations and significant material hardening in the later stages of deformation often pose a risk of cracking. To address this issue, crack stop incision was strategically designed at the four corners of the blank. The Coulomb friction model was adopted for describing the friction between the blank and dies, and the friction coefficient was set to 0.35 [24,25]. Figure 5a–c depict three common crack stop incision shapes. Implementing the corresponding designs for crack stop incision in the hot stamping process resulted in the distribution of equivalent plastic strain and the thickness of the formed component (highlighted by the red dashed lines in Figure 5). The findings indicate that the shape of the crack stop incision significantly influences the forming quality of the blank. Furthermore, the formed components display varying degrees of thinning in the connection area between the sidewall corner and the bottom of box. It is evident that the design presented in Figure 5a yields the best forming quality for the blank, while the maximum thinning rates for the formed components of the other two shapes reach as high as 34.5% and 44.2%, respectively, rendering those sheets unformable. Simulation results of the equivalent strain distribution reveal distinct differences in strain distribution at the side wall corner position for blanks with different shapes. In Figure 5d, the strain is primarily concentrated at the entrance fillet, indicating good forming quality at the connection between the sidewall corner and the bottom. In contrast, in Figure 5e,f, the equivalent plastic strain is highest in the connection area between the side wall corner and the bottom, suggesting this is a weak point in the forming process. This is attributed to the fact that, during the stamping process, the crack stop incision depicted in Figure 5a is the most conducive to material flow. However, at this juncture, the minimum thickness of the formed component is 1.586 mm. Typically, the thinning ratio of a titanium alloy sheet in hot forming should be controlled within 20%. Therefore, there is a perceived risk of rupture in the formed component, necessitating the further design of process parameters to ensure the high quality of the formed component.

#### 3.1.3. Determination of Forming Process Parameters

RSM (response surface methodology) was initially introduced by British statisticians G. Box and Wilson in 1951 [26], and it is mainly used to study how to carry out tests, modeling, and data analysis when the target response of a certain problem is affected by multiple variables. In the selection of process parameters for the hot stamping process, the use of RSM allows for a visual analysis and comparison of the effects of different factors on forming quality. Moreover, it can efficiently and accurately screen for the optimal combination of process parameters, significantly reducing simulation calculation time [27].

By analyzing the forming process of the box-shaped component, it is observed that the entrance fillet radius, blank-holder force, and friction coefficient have significant impacts on the forming quality. A larger entrance fillet radius facilitates easier material flow into the die during the stamping process. However, when the dimensions of the formed component are already determined, increasing the size of the entrance fillet radius facilitates an increase in the stamping depth, which poses difficulties for forming. When the blank-holder force is too large, the blank material pressed between the concave die and blank-holder is difficult to move, resulting in a high rate of thinning and the rupture of the formed part, whereas insufficient blank-holder force can lead to severe wrinkling of the blank and height wrinkling at four corners of the entrance fillet radius, obstructing the normal flow of the material and causing cracking in the weak regions of deformation. While the friction coefficient is a key factor affecting the ease of material flow inside dies during deformation, it works together with the recess die round angle and crimping force to affect the deformation of the titanium alloy blank during hot press forming.

Due to the fact that excessive parameters can decrease computational efficiency and negatively impact the accuracy of the response surface model, disrupting the optimization process, three key parameters were chosen as response variables: entrance fillet radius, blank-holder force, and friction coefficient. The minimum thickness R of the formed part is used as the response value. This is because the most severe thinning usually occurs at the corners of box-shaped parts. As a result, a larger R value indicates higher quality and better performance of the formed part; thus, the factor value corresponding to the largest R value is taken as the optimal combination of process parameters for forming. In the simulation process, the entrance fillet radius was set to 6 mm, 8 mm, 10 mm, 12 mm, and 14 mm; the blank-holder force was set to 1 kN, 6 kN, 11 kN, 16 kN, and 21 kN; and the friction coefficient was set to 0.25, 0.3, 0.35, 0.4, and 0.45.

To further investigate the impact of entrance fillet radius, blank-holder force, and friction coefficient on the forming quality and obtain the optimal process parameter combination for stamping titanium alloy box-shaped parts, this study used Central Composite Design (CCD) to design the experiments. CCD is the most commonly used second-order design in response surface methodology [28], suitable for experiments with multiple factors and levels where continuous variables are present. Compared to other RSM approaches such as Box–Behnken Design (BBD), this method offers better fitting of the response surface and greater flexibility, and it can eliminate missed runs or mismeasured responses [29,30]. Based on the numerical settings for the three key forming variables, the experimental design and results are presented in Table 2. In the model, the variables A, B, and C represent blank-holder force, friction coefficient, and entrance fillet radius, respectively.

The experimental results were analyzed by multivariate quadratic regression fitting to obtain the regression fitting equation of the minimum thickness value R of the part with the corresponding variables, as shown in Equation (1):

R = 1.62 − 0.0132A − 0.0368B + 0.0286C − 0.0061AB − 0.0219AC + 0.0051BC − 0.0764A^2^ − 0.0246B^2^ − 0.0771C^2^ (1)

In the fitting statistics, the value of adjusted R^2^ is 0.9334, which is higher than 0.9; the value of predicted R^2^ is 0.8003, and the difference with adjusted R^2^ is less than 0.2, indicating a high level of confidence in the predicted values. The variance analysis results of the model are shown in Table 3. The calculation results show that the F-value is 30.59, suggesting that the proposed scheme is highly significant. Two of the three response values have *p*-values higher than 0.05, and the *p*-value of the lack of fit is 0.2626, which is much higher than 0.05, indicating that the model is reasonable in the regression region [31]. According to the ANOVA results, the primary terms B and C as well as the secondary terms A^2^, B^2^, and C^2^ all have significant impacts on the response variable R. The coded equation is useful for identifying the relative impact of the factors by comparing the factor coefficients, and the effects of these significant factors on R are in the following order: C^2^ > A^2^ > B > B^2^ > C.

**Figure 6:**
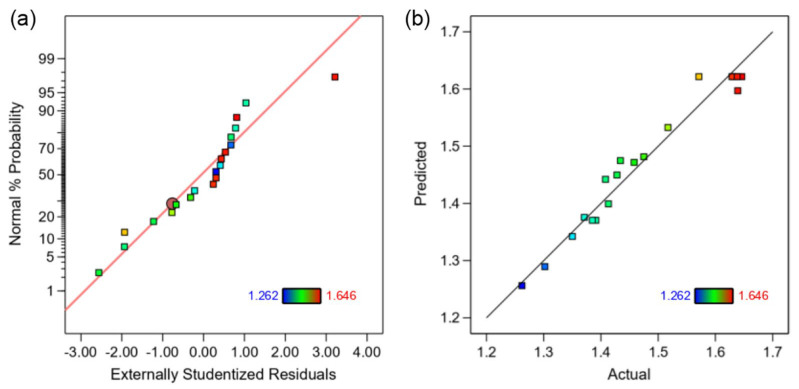
(**a**) The normal probability; (**b**) the predicted response values vs. the actual response values.

To further verify the accuracy of the fitted equations, diagnostic plots are generated as shown in Figure 6. Figure 6a shows the diagnostic plot of the normal distribution of residuals, and the data points are uniformly distributed on both sides of the diagonal line, manifesting that the fitting results obey a normal distribution. Figure 6b shows the distribution of predicted and real values, and the actual values are basically distributed in a straight line, showing that the predicted values basically match the actual values [32].

**Figure 7:**
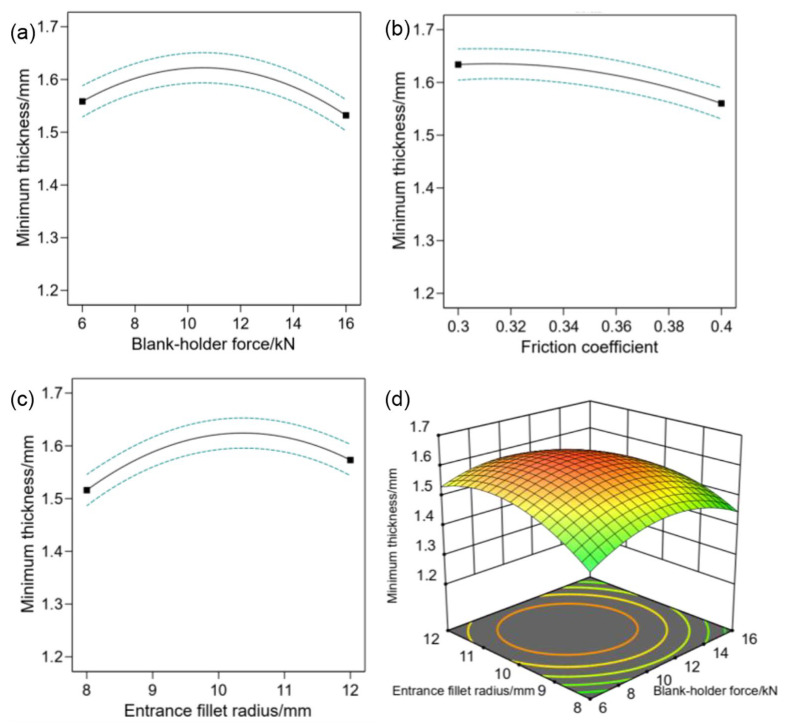
The effect of a single factor and two factor interaction on the R value: (**a**) A; (**b**) B; (**c**) C; (**d**) A–C.

To delve deeper into the individual factors influencing thinning, Figure 7a–c illustrate the impacts of entrance fillet radius, blank-holder force, and friction coefficient on the minimum thickness R of the components. From Figure 7b, it is evident that the minimum thickness of the formed component decreases with an escalation in the friction coefficient. This phenomenon is ascribed to the higher friction coefficient, which results in an augmented adhesive friction between the blank and dies. Consequently, it hinders the flow of the blank into the die gap during deformation, leading to more pronounced thinning during the final forming stages. Figure 7a,c demonstrate that as the blank-holder force and entrance fillet radius increase, the minimum thickness of the formed component initially rises before subsequently decreasing. Therefore, the interaction between blank-holder force and entrance fillet radius on minimum thickness can be predicted using response surface methodology, as shown in Figure 7d.

Through the optimization algorithm, the maximum value of response variable R is set as its optimal solution objective, and the best parameter combination is obtained: A = 10.591, B = 0.314, C = 10.347, and the minimum thickness of the formed component is predicted to be 1.638 mm.

### 3.2. Hot Stamping Experimental Verification

**Figure 8:**
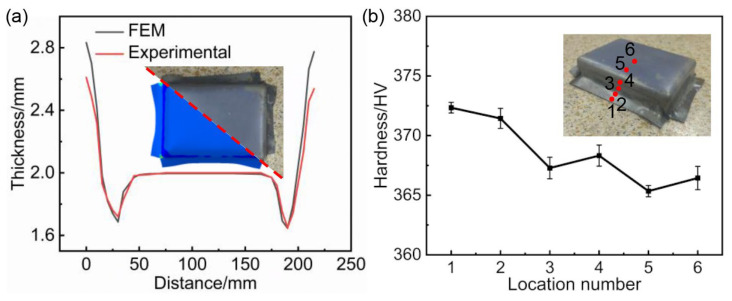
(**a**) Thickness and (**b**) hardness distribution of the formed part.

Based on the optimized process parameters obtained from the above procedure, a hot stamping experiment was conducted on a Ti6Al4V alloy sheet. The sheet was placed in a heating furnace, heated to 850 °C, and then held at this temperature for 2 min to ensure uniform temperature distribution throughout. After heating, it was quickly transferred to the room-temperature dies and then stamping was carried out at a speed of 50 mm/s. The formed component was cut along the section shown in Figure 8a using an EDM wire cutting machine (DK7750, Taizhou Borgman Machinery Equipment Co., Ltd., Taizhou, China). Along the cut section, we measured the thickness at intervals of 5 mm using a vernier caliper and compared the measurements with simulation results. The results demonstrate a close correlation between the thickness of the formed components and the simulation results, validating the accuracy of the simulation process. The thickness curve of the component shows some portions higher than the original thickness of the sheet. This is because wrinkling occurs at the entrance fillet of the sheet metal. However, since the entrance fillet and flange will be cut off in subsequent processing, it does not affect the forming quality of the box-shaped component. In order to obtain the hardness data at different locations of the formed component, six samples were cut sequentially from different locations of the formed component and tested for hardness, the sampling locations and sequence are shown in Figure 8b. Further analysis reveals variations in hardness at different positions of the formed components, with higher hardness in the flange section and relatively uniform hardness distribution in the sidewall and bottom of the box-shaped component.

**Figure 9:**
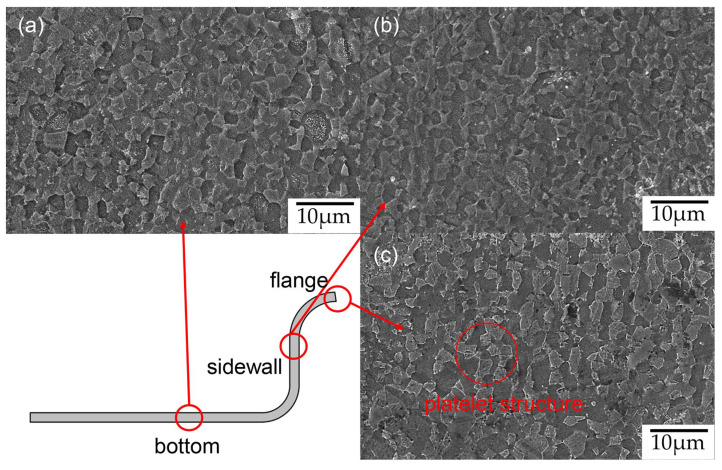
Microstructure of (**a**) the bottom of the formed part; (**b**) the sidewall; (**c**) the flange.

To investigate the reasons for the local hardness variations in parts and further study the evolution mechanism of the microstructure during the hot stamping process of Ti6Al4V alloy, specimens were taken from the flange, sidewall, and bottom of the formed component, as illustrated in Figure 9. After grinding and polishing, the specimens were etched using a solution composed of 7% HF, 13% HNO_3_, and 80% H_2_O. Subsequently, scanning electron microscopy (SEM) tests were conducted to observe the microstructures of these three specimens, as shown in Figure 9. The protruding parts represent the β phase, while the α phase appears as a downward concave morphology under the action of the etching solution. A comparison reveals significant microstructural differences in various parts of the formed component, attributed to the uneven deformation and temperature changes across different areas of the sheet during the hot stamping process. The grains in Figure 9b are noticeably finer than those in Figure 9a,c, primarily because deformation mainly occurs in the sidewall section. Current research indicates that the thermal deformation process of titanium alloys can trigger dynamic recrystallization mechanisms, leading to grain refinement. Furthermore, as the strain increases, the driving force for recrystallization intensifies, resulting in a higher proportion of recrystallized grains and enhanced grain uniformity [33]. Fine grain strengthening is one of the main strengthening mechanisms for titanium alloys. The grain refinement and grain boundary strengthening induced by dynamic recrystallization significantly enhance the strength and hardness of the material [34,35]. This explains why the hardness of the sidewall section of the formed part is slightly higher than that of the bottom.

**Figure 10:**
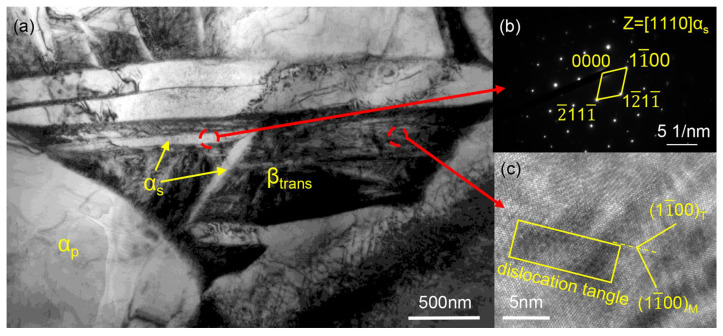
TEM micrograph of Ti6Al4V alloy after hot stamping: (**a**) BF image; (**b**) SAED corresponding to secondary α phase; (**c**) HRTEM image of typical transformed β phase.

A comparison of Figure 9a,c reveals that platelets embedded in the transformed β phase are found to be present in Figure 9c. Transmission electron microscopy (TEM) tests of this type of structure (Figure 10) show that the platelets are a secondary α phase embedded in the nano-scaled transformed β phase (Figure 10b). A high density of dislocation tangle zone and twin structures were found in the transformed β phase (Figure 10c). It is generally believed that the secondary α phase appears in the hot stamping process at a heating temperature above 900 °C [36], resulting in an increase in the hardness of the sheet metal, which makes the surface of the component more prone to cracking during the stamping process, significantly reducing the forming quality [37]. However, since the flange section is mainly subjected to blank-holder force, which is the part subjected to the highest pressure from dies in whole formed part, its cooling rate is the fastest [38], resulting in the secondary α phase appearing under deformation conditions at a heating temperature of 850 °C, indicating that a higher cooling rate is an important factor for the generation of a secondary α phase during the hot stamping process. Zhu et al. [39] preformed some heat treatment experiment where TA15 titanium alloy samples were soaked at 960 °C for 30 min and then cooled at different rates. They found that increasing the cooling rate promoted the growth of a secondary α phase in the titanium alloy. This indicates that cooling rate is an important factor influencing the occurrence and growth of a secondary α phase. Therefore, in the design of hot stamping processes for titanium alloys, attention should be paid to the reasonable design of forming process to prevent excessively high cooling rates in localized areas of the sheet. For parts requiring the removal of flanges, higher blank-holder force can be applied to the sections that will be cut off, but when the flange section is necessary in the forming parts, the blank-holder force should be appropriately designed in order to avoid the unfavorable microstructural effects caused by the phase transformation. 

## 4. Conclusions

In this paper, the high-temperature mechanical properties of a Ti6Al4V alloy were investigated. Based on this, the hot stamping process of a Ti6Al4V alloy box-shaped part was simulated and optimized using ABAQUS 2020 finite element software. The accuracy of the numerical simulation was verified through thermal stamping experiments, and the microstructural evolution of the hot stamping process of Ti6Al4V alloy was revealed, which provides effective guidance for the process improvement and parameter optimization of the hot stamping of titanium alloy sheets. The main conclusions are as follows:

1. The thermomechanical properties of Ti6Al4V alloy at different temperatures (400 °C, 500 °C, 600 °C, 700 °C, 800 °C) and different strain rates (0.1 s^−1^, 0.05 s^−1^, 0.01 s^−1^) were investigated, which showed that with the rise in deformation temperature and the decrease in strain rate, the tensile strength of the material decreased, while the elongation increased, indicating the plasticity of material was enhanced. The tensile strength of the material at 400 °C was higher than 808 MPa, which was close to its mechanical properties at room temperature, indicating that the hot stamping process of the blank should be above this temperature.
2. The performance analysis revealed that under the conditions of a blank-holder force of 10.591 kN, a friction coefficient of 0.314, and an entrance fillet radius of 10.347 mm, optimal performance for titanium alloy box-shaped components in hot stamping was achieved. The simulation results were validated through hot stamping experiments, and the results showed good agreement.
3. The research on the microstructural evolution of the formed parts indicated that in hot stamping formation at a heating temperature of 850 °C, a higher cooling rate induces the precipitation of a secondary α phase, resulting in higher local hardness in formed parts. The sidewalls of the box-shaped parts with higher deformation undergo grain refinement strengthening and were therefore of slightly higher hardness than the bottom of the box-shaped parts. The formed box-shaped parts exhibited a uniform and fine microstructure overall, demonstrating high forming quality.

## Figures and Tables

**Table 1:** Chemical composition of Ti6Al4V alloy.

Element	Ti	Al	V	Fe
Content/wt%	Bal.	6.0	3.92	0.19

**Table 2:** Experimental design and results.

Run	A	B	C	R
	Blank-Holder Force/kN	Friction Coefficient	Entrance Fillet radius/mm	Min Thickness (mm)
1	11	0.35	10	1.571
2	15	0.4	12	1.413
3	11	0.35	6	1.262
4	16	0.3	8	1.458
5	11	0.45	10	1.428
6	6	0.4	8	1.391
7	11	0.35	10	1.638
8	11	0.35	10	1.635
9	11	0.35	14	1.385
10	11	0.25	10	1.639
11	16	0.4	8	1.371
12	6	0.3	12	1.517
13	16	0.3	12	1.434
14	6	0.3	8	1.408
15	11	0.35	10	1.646
16	6	0.4	12	1.475
17	11	0.35	10	1.629
18	1	0.35	10	1.350
19	11	0.35	10	1.631
20	21	0.35	10	1.302

**Table 3:** Analysis of variance (ANOVA) for the maximum thickness.

Source	Sum of Squares	df	Mean Square	f-Value	*p*-Value
Model	0.2860	9	0.0318	30.59	<0.0001
A	0.0028	1	0.0028	2.68	0.1328
B	0.0217	1	0.0217	20.87	0.0010
C	0.0131	1	0.0131	12.56	0.0053
AB	0.0003	1	0.0003	0.2889	0.6027
AC	0.0038	1	0.0038	3.68	0.0839
BC	0.0002	1	0.0002	0.2022	0.6625
A^2^	0.1469	1	0.1469	141.41	<0.0001
B^2^	0.0152	1	0.0153	14.61	0.0034
C^2^	0.1493	1	0.1493	143.73	<0.0001
Residual	0.0104	10	0.0010		
Lake of fit	0.0067	5	0.0013	1.82	0.2626
Pure error	0.0037	5	0.0007		
Cor total	0.2964	19			

## Data Availability

Data are contained within the article.
